# An Optimal, Power Efficient, Internet of Medical Things Framework for Monitoring of Physiological Data Using Regression Models

**DOI:** 10.3390/s24113429

**Published:** 2024-05-26

**Authors:** Amitabh Mishra, Lucas S. Liberman, Nagaraju Brahamanpally

**Affiliations:** 1 Department of Cybersecurity and Information Technology, Hall Marcus College of Science and Engineering, University of West Florida, Pensacola, FL 32514, USA; 2 Department of Computer Science, Hall Marcus College of Science and Engineering, University of West Florida, Pensacola, FL 32514, USA

**Keywords:** electrocardiogram (ECG/EKG), arterial pressure (ART), Internet of Medical Things (IoMT), regression

## Abstract

The sensors used in the Internet of Medical Things (IoMT) network run on batteries and need to be replaced, replenished or should use energy harvesting for continuous power needs. Additionally, there are mechanisms for better utilization of battery power for network longevity. IoMT networks pose a unique challenge with respect to sensor power replenishment as the sensors could be embedded inside the subject. A possible solution could be to reduce the amount of sensor data transmission and recreate the signal at the receiving end. This article builds upon previous physiological monitoring studies by applying new decision tree-based regression models to calculate the accuracy of reproducing data from two sets of physiological signals transmitted over cellular networks. These regression analyses are then executed over three different iteration varieties to assess the effect that the number of decision trees has on the efficiency of the regression model in question. The results indicate much lower errors as compared to other approaches indicating significant saving on the battery power and improvement in network longevity.

## 1. Introduction

Healthcare demands are rising the world over, and so are the costs involved. One of the biggest challenges being faced by the world healthcare systems is the growth of the world population with a sizable number of geriatric patients that form a high percentage of the total population. This growth inevitably adds to the already mounting costs for healthcare, owing to the demand for significant increase in resources. The rise in healthcare costs and the longer life expectancy are directly connected to this increase in the number of geriatric patients, which form two of the biggest problems that the global health scenario faces today. According to several studies, global healthcare spending in developed and developing nations is anticipated to reach 4–11% of their respective GDP’s by 2030 based on current trends [1,2,3]. The world economy has already started facing the adverse effects caused by these challenges.

To meet such high increase in healthcare demands, medical professionals are increasingly adopting new wireless technologies to monitor their patients’ conditions without necessitating the patients’ physical presence at a medical facility or constraining them to beds. Detecting a disease proactively at an early stage can mitigate the costs of a potential treatment by treating or controlling the disease early in its cycle before it can cause serious physiological damage to the patient. Research has demonstrated that the majority of afflictions and diseases can be avoided with early detection in the initial stages of the health issue. This fact suggests that future health care systems should guarantee proactive wellness management with a change in approach towards medical practices. A potential solution to proactive and more affordable health care systems may be provided by wearable monitoring systems, which have appeared in the healthcare market in recent years. These systems can help with early detection of abnormal conditions and significantly raise the state of public health in the community at large, and more prominently in the geriatric population.

### 1.1. The Internet of Medical Things (IoMT) Paradigm

In recent decades, advancements in the semiconductor industry have led to the miniaturization and cost reduction necessary to produce computers smaller than a pinhead, yet powerful enough to handle necessary processing tasks, and inexpensive enough to be viewed as disposable. The years ahead will undoubtedly witness a further decrease in size with an enhancement in processing capability, as novel categories of computers or smart devices emerge in each successive decade [4]. Likewise, progress in sensor development, wireless communication and energy-storage technologies has led to the realization that a fully pervasive network using wireless sensors deployed for health monitoring has almost come to fruition [5]. Micro-sensors, measuring just a few millimeters in size, equipped with onboard processing and wireless data-transfer capability, form the fundamental building blocks of specialized sensor networks that have been in operation for above ten years now [6,7].

**Figure 1:**
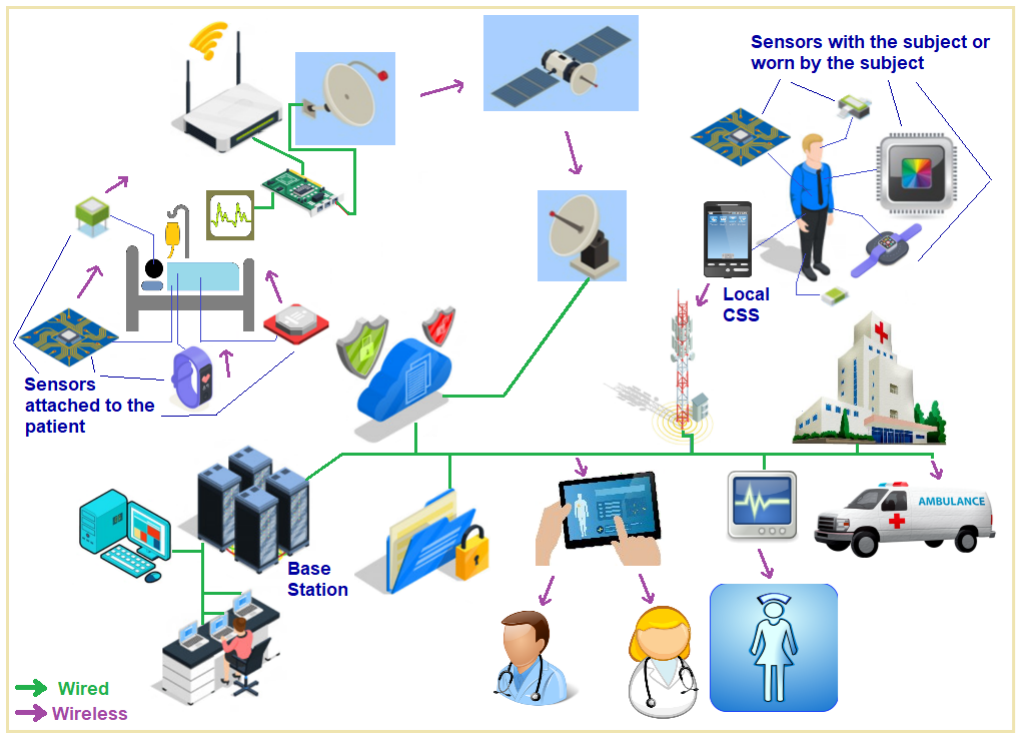
An Internet of Medical Things (IoMT) network.

These developments have resulted in the formulation of the paradigm of the Internet of Things (IoT) that makes use of mobile technologies such as wearable and implantable sensors [8]. Such sensors are called Internet of Medical Things (IoMT) nodes, to create an information-relay system to transmit important physiological and bio-kinetic signals over cellular networks as mentioned by Crosby et al. [9]. Figure 1 shows the use of Internet of Medical Things which is a path breaking concept that uses low power, short-range sensor nodes to sense and monitor bio-kinetic and physiological parameters of a living subject—a human being or an animal. These sensor nodes then transmit their data using wireless link hops over a network to a central unit, called a local base station that could be a standalone device or a smartphone running an app for this purpose. Data transmission to a base center through this sensor network up to the local base station may involve a single hop or multiple hops through additional IoMT nodes. The local base station collects these data, performs some immediate processing required on it and then transmits the semi-processed data over the internet to the emergency medical team for any urgent needs of the subject, to the personal physician, a specialist doctor, a surgeon or to the nursing station. The data can be simultaneously relayed to a data center for further analysis and storage. The network can employ a variety of protocols and network types starting from near field communication to Bluetooth, Wi-Fi, the cellular data network, or satellite links—or a combination of these for transmission to the various end data consumer parties.

### 1.2. An IoMT Network with High Availability and Redundant Network Design

A network created using IoMT nodes envisions a human-centric application of wireless technology for individualized telehealth and telemedicine, which eliminates the need for patients to remain bedridden, hospitalized or under the constant supervision of medical personnel or doctors. In addition to tracking patients’ and athletes’ physiological and biokinetic parameters, the idea can also be applied to save lives, especially for the astronauts, first responders, divers, firefighters and personnel who work in dangerous environments. The demand for incorporating IoMT systems into the upcoming telecom infrastructure and information technology has increased vastly in proportion to the rise in healthcare costs globally.

By implementing proactive concepts, IoMT networks can increase healthcare efficiency and reduce costs.

The key to the adoption of the IoMT is the deployment of wireless physiological sensors for creating an IoMT network as an important subset within the IoT paradigm given by Mishra and Agrawal [10]. IoMT takes advantage of low power, small radio range sensor nodes for sensing the physiological or bio-kinetic link hops over a network. IoMT promotes proactive concepts by not only monitoring vitals from various physiological organs and body parts, but also by providing proactive cardiovascular monitoring that is key to identifying potential heart ailments before they can take effect. Other life-threatening diseases can be preemptively diagnosed, most notably different strains of cancer that can be diagnosed or predicted for the start of early treatment which can reduce the death rates caused by cancers as mentioned by Kim et al. [11].

**Figure 2:**
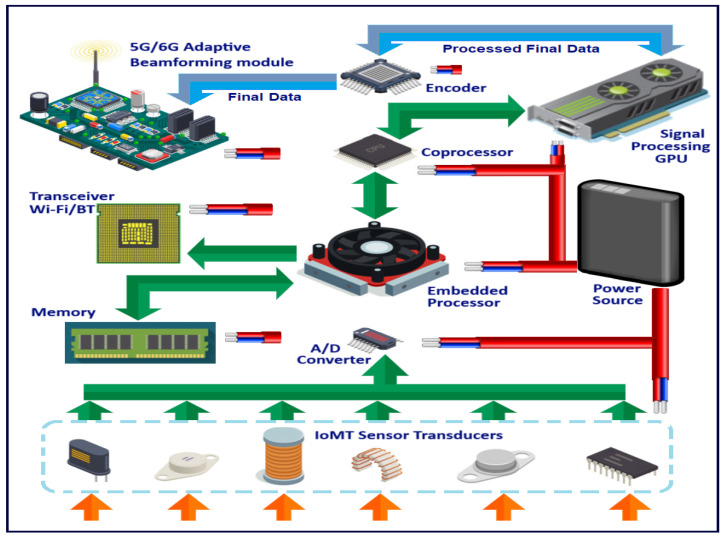
IoMT data collection, processing and transmission through data/cell phone networks.

The IoMT architecture proposed by the authors and tested in a previous work is specifically tailored for the wireless networking of implantable and wearable body sensors. Its main objective is to establish a benchmark for the advancement of a unified methodology in pervasive monitoring. Figure 2 depicts the fundamental idea behind this architectural design. The subject is equipped with multiple physiological sensors that are connected to their body. Each sensor is also linked to its own small, embedded processor that has its own memory. A single battery powers all the components shown in the figure, and each component contributes to the battery drain, with the maximum battery expenditure incurred in transmission of data samples. The system has capabilities to communicate processed sensor data using multiple wireless transmitters for a variety of wireless network types such as Wi-Fi, Bluetooth and the cellular phone and data 5G/6G network. The performance and processing capabilities of a sensor can also be enhanced by additional processor/s for generating an optimized data output to be sent over to the base station for data collection, curation and analysis. These sensors are by and large, powered by a battery. Together, they create an “IoMT node complex” that can seamlessly integrate with home, office and hospital environments. The IoMT node guarantees precise data capture from the sensor it is linked to, performs basic data processing and subsequently sends this information to a Local Processing System over a wireless link. The information gathered by the sensors is collected by the processing unit where it undergoes additional processing using one or more processors. The data from multiple sensors are then combined before being sent wirelessly to a central monitoring server through a wireless LAN or the cellular (5G/6G) network [12].

Our suggested design, as illustrated in Figure 2, includes improving the body sensor nodes described in [13] by incorporating a coprocessor. This additional microcontroller aids in simplifying data logging, processing and temporary storage of data samples from the sensor nodes. The microcontroller is equipped with a wireless extension that enables the sensor node to communicate with the CSS or similar devices via 6G or other commercial voice/data networks, in addition to the radio for sensor networks. The extended architecture refers to the 6G extension for the sensor node, which incorporates dedicated channels for communication and processing of sensor data from the IoMT network. This addition is based on the assumption that the 6G standards will include provisions for such functionalities. An IoMT network could greatly benefit from this improvement as it would allow the sensor nodes to establish communication with a CSS, which could potentially be a smartphone, eliminating the necessity for a separate CSS device.

## 2. Significance of IoMT Sensor Data and Their Transmission and Processing

By implementing proactive concepts, IoMT networks can increase healthcare efficiency and reduce costs. IoMT networks have a significant hidden capability to transform the future of patient monitoring by eliminating the need for expensive in-hospital patient monitoring as well as by diagnosing several life-threatening diseases [14]. According to estimates, the number of new cancer cases could rise to 29.5 million by 2040, with the increase in mortality to 16.4 million [15,16]. With no need for a biopsy, early tumor diagnosis can be influenced by IoMT-based cancer cell monitoring, which also provides timely analysis for early detection and treatment.

### 2.1. IoMT Networks for Monitoring of Cardiovascular Data

In addition to monitoring vital signs, seizures, or organ implants for a variety of medical monitoring applications, proactive cardiovascular monitoring is a very important use of IoMT systems. This can be achieved by the IoMT sensor nodes that spot potential heart conditions before they occur and report them promptly to the medical team along with any alarms generated.

Cardiovascular disease is the significant other leading cause of death, accounting for close to 32% of fatalities globally [17,18]. This is why the authors chose to create an optimal and power efficient IoMT monitoring model for cardiovascular signals in their research.

The ECG sensors used for identifying ischaemic heart disease measuring cardiac output can either be implantable or wearable sensors, while the other option of finding out cardiac output by detecting Troponin and creatine kinase levels can be performed through implantable biosensors. In addition to measuring blood pressure, mechanoreceptors can also be used to sense the heart rate and cardiac output, enabling the detection of cardiac arrhythmias or heart failure [19]. Significant advances have also been made in the field of coronary surgeries through micro-robots [20].

Numerous recently developed sensors, designed for both wearable and implantable purposes, have been introduced due to advancements in mechanical, electrical, biological and chemical sensor technology. The capability of pervasive patient monitoring is illustrated by the following examples, regardless of the broad spectrum of applications that these sensors can be utilized for. Pin pricks and blood draws for routine invasive testing can now be replaced with implantable sensors or wearable patches [21].

Given that biosensors are now being utilized in closed feedback loop systems that ultimately guide the administration of treatment, the importance of reliability cannot be overstated in these applications, as the lives of individuals depend on them. It has been discovered that implanted biosensor arrays can offer a higher level of accuracy compared to a single, independent sensor [22].

Alongside simultaneous progress in advancements in sensor production, MEMS technology and nano-engineering techniques offer the potential to develop significantly smaller implantable and attachable sensors. An example are the ones used for blood glucose measurement through fluorescent optical sensors which have much smaller form factors as compared to the ones previously achievable [23]. Despite the continuous reduction in size and power requirements of these sensors, they still rely on electrical power and will probably be powered by a battery source for a considerable time in the future.

### 2.2. Energy Supply and Demand in IoMT Networks

IoMT networks must prioritize power consumption as it directly impacts both the battery size and the duration that the sensors can remain in action. The battery size utilized for storing the necessary energy typically plays the most significant role in determining the overall size of the sensor device, impacting both its dimensions and weight. The significance of these factors extends beyond just the implantable sensors to also encompass the external sensor settings, as they play a crucial role in determining the level of concealment and ubiquity of the sensors. Wireless communication over short distances can use ultra-wideband radio technology that offers high data speeds and minimal power usage [24]. Self-organizing networks offer the benefit of decreasing energy usage by eliminating superfluous or redundant nodes, consequently extending the longevity of the system. Although small, high-density batteries are commonly used for wearable and implantable sensors due to their convenience, there has been ongoing research on alternative methods to generate the necessary power.

In order to achieve this goal, a strategy is being developed to improve battery life by reducing battery consumption through increased utilization of power scavenging from on-body sources like vibration and temperature, which would benefit implantable sensors immensely [25,26]. Currently, a significant amount of the electricity utilized by biosensors is allocated to the measurement circuit. Within an IoMT network, the wireless communication connection is expected to be the primary power consumer. The successful development of IoMT networks relies heavily on the key aspect of developing low-power wireless data paths. The practical deployment of IoMT networks depends greatly on minimizing the power consumption of the radio transceiver [27,28].

### 2.3. The Energy-Fidelity Tradeoff in IoMT Sensors

The tradeoff between energy and fidelity in IoMT networks is one of the key performance and design issues for these networks. The IoMT networks must convert and transmit the sensed parameters into valuable information with an acceptable and suitable level of fidelity while using minimal energy. Selective processing of various physiological data samples is a technique that can be used for improving the energy efficiency for optimal operation and the lifetime of the battery powered sensors used for creating such networks while not compromising on the data fidelity to an extent that the medical diagnosis or proper and accurate interpretation of medical data would be impacted.

Increasing the lifetime of the IoMT sensor nodes and, by extension, that of the IoMT networks, is a constant challenge because of the restriction of small sensor node size and even smaller batteries. IoMT network nodes are comparable to their wired instrumentation network counterparts, but they are smaller and have lower battery capacities, which puts them at a severe disadvantage. The sensor nodes must continue gathering samples of data to relay to the base station, while being powered off a battery of some kind that needs to be replenished or replaced.

The energy-fidelity trade-off is inherent to an IoMT network if sensor data rates are throttled, which means that higher data rates obviously mean a higher drain on the battery which provides more reliable data. Simply put, the relatively small size of the sensor nodes and its short battery life acts as a hindrance in the effective transmission of all physiological data on a continuous basis without interruptions when powered by a battery. Consequently, the signal processing methods involved must exclude some of the sample data from transmission and then reliably predict those missing values upon reception of the signals by the coordinating sink station (CSS) for the collection and consolidation of IoMT data.

### 2.4. Recent Techniques for Improving the Energy Efficiency in IoMT Networks

Ashween et al. [29] discuss an energy-efficient data-gathering technique for Wireless Sensor Networks (WSNs) that utilizes a mobile sink node for improved network lifetime. It introduces an embedded routing protocol based on optimal mobile sink node selection, which is enhanced by an Oppositional Grey Wolf Optimization (OGWO) algorithm. The study aims to reduce energy consumption and delay in WSNs, with performance compared against existing routing protocols to demonstrate efficiency.

The continuous, long-lasting and reliable energy supply for implanted devices is becoming increasingly crucial due to the advancements in miniature devices for biomedical applications.

Various options for energy harvesting power sources encompass heat, solar energy, air or fluid movement, pressure differentials, vibrations, chemical or radioactive reactions and a diverse array of biological sources. It is feasible to extract power from low-frequency body motion for implants, such as in the case of implants. The capability of utilizing linear and nonlinear energy harvesters to harness the energy from heart-beat vibrations for powering pacemakers has been demonstrated by Inman and Karami [30]. These are robust to changes in heart rate and are designed based on the features of heartbeats.

In addition, Yeatman et al. have proposed various low-frequency body motion power harvesters. They have recently shown how stepwise microactuators can be operated by ultrasonic transfer [31].

### 2.5. Micro-Fuel Cells for Efficient Powering of IoMT Sensors

Creation of micro-fuel cells, which can be used in implantable sensors to reduce the size of the control supply and increase the battery’s and the sensor’s lifetime is another technique. Fuel cells possess several characteristics that make them extremely appealing for portable power generation. These include their remarkable energy efficiency and density, coupled with their capability to be refueled swiftly [32,33,34].

Solid-oxide fuel cells [35] and polymer-electrolyte direct methanol [36] are two examples of alternative technologies proposed for portable applications, aiming to replace Lithium ion batteries. Another approach entails the implementation of direct alcohol-based fuel cells, whereby the chemical energy present in the fuel/alcohol is electrochemically transformed into electricity. This method boasts a superior energy density with very low pollutant emissions [37].

Additional ramifications of implementing IoMT networks involve the recognition of the enduring outcomes stemming from their impact on the human body, specifically regarding implantable sensors. The materials and manufacturing process employed in building IoMT network nodes, along with their battery supply and the method of wireless data transmission utilized, are anticipated to be regulated by these outcomes. In an IoMT network consisting of multiple nodes, the presence of numerous energy-depleted nodes can have a negative impact on the environment. Hence, it is essential for these nodes to be reusable or biodegradable in order to mitigate any potential harm to the environment.

### 2.6. Newer Networks with Low-Energy Requirements, Energy Harvesting and Cloud Processing

In [38], Puspitaningayu et al. gathered and processed the data from an embedded system using Bluetooth low energy links and a cable connection. Wagner et al. [39] and Nandkishor et al. [40] proposed similar systems for Android operating system-based hosts with some variations. The data were then transmitted over short range wireless links to the smartphone for analysis and interpretation. Ogunduyile et al. used a method [41] that involved transferring the IoMT data to a Medical Health Server for examination over a GPRS/Internet connection. Using a custom sensor network for data logging, processing and analysis, Baviskar and Shinde [42] sent the findings to the sink station via Bluetooth links. The data were beamed up to a server over a 5G with MIMO link in the system proposed by Al-Asadi et al. [43].

Yang Wang [44] proposes an integrated energy-efficient strategy for IoT devices, aiming to reduce energy consumption while also capturing new energy through RF energy harvesting. One aspect involves designing a node sampling scheduling algorithm based on matrix completion to minimize data sampling while maintaining data integrity. Additionally, an adaptive RF energy management strategy is introduced, enabling automatic switching between wireless information transfer and wireless energy transfer modes to enhance energy efficiency. Overall, this integrated solution aims to prolong the battery lives of IoT devices by decreasing energy consumption and providing continuous energy through RF transmission.

In [45] the Internet of Medical Things (IoMT), a crucial component of the medical sector’s Internet of Things (IoT), enhances healthcare quality by enabling real-time monitoring and reducing medical expenses. IoMT benefits from edge and cloud computing, which offer increased computational and storage resources near terminals to meet low-latency demands for computation-heavy tasks. However, offloading services from health monitoring units (HMUs) to edge servers increases energy consumption, but leveraging artificial intelligence (AI) like Asynchronous Advantage Actor-critic (A3C) can optimize resource allocation, as demonstrated in the authors’ proposed energy-aware service offloading algorithm, ECAC, tailored for an end-edge-cloud collaborative IoMT system.

### 2.7. Achieving Energy Efficiency by Throttling the Rate of Sensor Data

Hardly any of the approaches discussed above consider the energy expenditure and economizing on the power usage in sensors while achieving the objectives through sensor data reduction while still meeting the QoS requirements specified for the specific physiological parameters. In a previous work connected to the present article, Mishra and Agrawal [10] introduced a new architecture that captured and transmitted cardiac parameters using IoMT sensors to a local sink station that compressed, processed and sent the data over cellular networks to a remote central base station for demodulation and decompression, while also focusing on reducing the amount of energy used. The physiological parameters are continuously monitored by the IoMT nodes and corresponding large numbers of data samples are captured and transmitted to the base station and beyond. However, the quantity of samples taken does not account for the frequency and type of physiological parameter variations. By using signal processing techniques that involve excluding some sample data from transmission and recreating missing samples using prediction, the authors have also attempted to reduce the amount of data in this paper in order to address the energy-fidelity trade-off.

The study by Callens et al. presents random forest and gradient boosting trees as alternative algorithms to neural networks for improving sea state forecast accuracy, comparing their performance with neural networks for the first time [46]. Results demonstrate that both random forest and gradient boosting trees achieve lower root mean square error (R(MSE) values, by 10% and 20% respectively, indicating their potential for enhancing forecast accuracy. This study develops gradient boosting and random forest models to forecast Japan’s real GDP growth from 2001 to 2018, outperforming benchmark forecasts [47]. By employing cross-validation for hyperparameter optimization, the gradient boosting model demonstrates superior accuracy compared to the random forest model, advocating for increased adoption of machine learning in macroeconomic forecasting. This study employs gradient boosting regression and random forest models to predict net ecosystem carbon exchange (NEE), demonstrating superior performance compared to other state-of-the-art models [48]. The identified important variables influencing NEE include global radiation, photosynthetic active radiation, minimum soil temperature and latent heat, with the proposed methodology showing promise for ecosystem stability evaluation, environmental restoration and climate change analysis. Encouraged by the results from applications in other domains discussed in these articles, the authors prepared the data used in previous works to subject to application of these newer techniques to save on the energy usage in IoMT networks.

## 3. Prominent Challenges for IoMT in Cardiovascular Applications

The QoS parameters for wireless transmissions of cardiovascular data are the most stringent in any kind of wireless sensor networks, which is why the authors chose to test their proposed scheme on IoMT sensors for sensing and relaying cardiovascular data.

Heart rhythm abnormalities, also known as arrhythmias, are commonly encountered in medical settings, impacting nearly 4% of individuals over the age of 60, with the prevalence increasing to around 9% in those over 80 years old [49]. Up to 10% of individuals who have reached the age of 65 years are impacted by heart failure [50]. The patients often seek medical advice due to the early signs of atrial fibrillation arrhythmias, which typically include fatigue and palpitations [51]. It is of utmost importance to address the potential long-term complications associated with tachycardia, such as cardiomyopathy and stroke, in order to prevent further deterioration of the patient’s condition [52]. Potential bleeding complications resulting from anticoagulant therapy contribute to an increase in mortality among elderly patients, along with various other risk factors [53]. It is highly desirable to continuously and extensively monitor the heart rate and other heart parameters of various patients, especially the elderly [14].

### 3.1. Important Physiological Measurements Involving Cardiac Activity

The systemic arterial pressure (ART) is a key vital sign that reflects the pressure of the blood circulating in the large arteries, and it is typically measured in millimeters of mercury (mmHg) within the systemic circulation. The value of the parameter changes with every heartbeat as the heart pumps, and it is determined by the cardiac output and total peripheral resistance. The arterial walls are subjected to systematic stress from varying levels of arterial pressure. The level of arterial pressure is directly correlated with arterial flexibility, peripheral vascular resistance and cardiac output [54]. It is crucial for the individual’s physique to possess the ability to adapt to sudden shifts in arterial pressure, and for the individual to seek medical treatment or make necessary lifestyle changes to address long-term fluctuations. Regulation of arterial pressure is essential for maintaining an adequate pressure level that allows for proper perfusion of organs and tissues, without reaching harmful levels. Another important parameter of the heart activity is the well-known ECG Lead-II signal that the physicians and heart surgeons use as the basic indicator of the activities of the four valves of the heart and their proper operation. A regular monitoring of the ECG signal also helps in identification of atrial fibrillation and other arrhythmias. It can initiate a prompt patient treatment for avoidance of complications such as rapid heart rate causing tachycardia and pump failure due to cardiomyopathy. Pervasive and continuous monitoring of the ECG signal is possible with IoMT sensor networks today.

### 3.2. Reducing IoMT Data without Compromising the QoS Requirements and Their Transmission

Whereas many studies have attempted to establish IoMT networks of their own [37,38,39,40,41,42], the approach in [55] used digital modulation to transmit the data first as text messages, and then as voice coded signals over regular voice channels to mitigate against potential inter and intra-network interference issues. Their study relied on sets of 3600 measurement samples covering 10 s of sensor data that included Electrocardiogram lead II (ECC/EKG) and Arterial Pressure (ART) signals compressed with delta modulation and its effect then transmitted to a smartphone that functioned as the CSS. The author’s subsequent work improved the sensor network design covered in [10] by giving IoMT the ability to communicate across commercial wireless voice/data networks. By complementing the solutions suggested by Jamthe et al. [56], the enhanced design’s benefit was helpful in reducing inter-IoMT and intra-IoMT interference problems which can increase the data rate requirements because of retransmission of data lost due to interference.

The architecture suggested by the authors can provide medical data updates via phone calls or brief texts to the right healthcare providers in the event that an emergency arises for a human patient under IoMT monitoring. Despite not being able to transmit continuous IoMT monitoring data, these updates can still support healthcare professionals in making early diagnoses, preparing for treatments and deciding on appropriate actions for patients.

For this research article, the authors have chosen to replicate the experiment on the same physiological data as described above but using two regression models that rely on decision trees to produce more accurate prediction sets at the cost of requiring more energy from the part of the CSS [57]. Decision trees represent a more sophisticated regression mechanism for predicting missing values based on machine learning algorithms [58]. Wozniakowski et al. [59] have revised gradient boosting in a way that allows it to produce a series of enhancements to a model that is not constant, potentially incorporating previous knowledge or a deeper understanding of the data-generation process. They suggest an alternative multi-target stacking method that enhances the approach to the context of multi-target regression. Jumin et al. [60] used both linear regression, neural network and the gradient boosting regression, what they refer to as the ‘Boosted Decision Tree’, to predict ozone concentrations and found that the latter outperformed the former two models at every iteration of their experiment. The consequence and practicability of this approach is discussed further in Section 7.

## 4. The Use of Decision Trees and Regression to Handle IoMT Data Reduction

Decision trees are used as predictors and classifiers; that is, they are computational constructions that attempt to predict or “classify” a data point by first asking a series of questions, treated as conditional statements, about the features of said data item prior to reaching a decision as put forth by Gupta et al. [61]. The algorithm is best visualized as a tree that starts at the root node and moves downwards according to the input features until the user reaches a leaf node, that is, a decision. Popular regression algorithms make use of an aggregation from many decision trees to produce more accurate predictions than a single decision tree, but it is how the aggregation occurs that distinguishes one regression model from another.

For our tests, we selected two regression algorithms with which to conduct our investigation: random forest and gradient boosting regressions. By ‘forest’, it is implied that the former uses an array of decisions on trees to predict its values. However, contrary to the latter, forest regression takes the aggregate sum of all decision trees to produce a single result. Gradient boosting borrows the concept of a forest but takes a different cumulative approach by applying the MART (Multiple Additive Regression Trees) algorithm whereby at each step the loss of function is defined to measure the error in order to correct the next tree until the optimum tree is reached according to Jumin et al. [60]. This process of building one to improve the deficiencies of its immediate predecessor is referred to as ‘boosting’. The difference between the two models, therefore, lies at the time of aggregation: whereas random forest trees will conduct its aggregation only after all of the decision trees have been executed, gradient boosting conducts its aggregation after each individual decision is made.

### 4.1. Decision Trees and Related Errors in Prediction

A decision tree is a hierarchical structure used for regression or classification tasks, with nodes making decisions based on specific functions and leaves providing final outcomes. A decision tree is a supervised learning algorithm used for classification and regression tasks. It has a hierarchical structure with a root node, branches, internal nodes and leaf nodes, which represent outcomes [62].

Decision tree regression, performed to estimate values like SM, offers advantages such as flexibility in handling various response types, simplicity in interpretation and validation through statistical tests. However, it can be unstable due to small variations in data and prone to overfitting, requiring careful parameter tuning to mitigate these issues. Despite its drawbacks, decision tree regression remains a reliable model choice when its advantages are leveraged while addressing its limitations. They used *MSE* (Mean Squared Error), *MAE* (Mean Absolute Error) and *R*^2^ (*R*-squared) as the evaluation metrics to arrive at a good fitness function [63].

The Mean Squared Error (*MSE*) is defined as:

(1) $$\mathrm{MSE}=\frac{1}{n}\sum_{i=1}^{n}{\left(Y_{i}-\hat{Y_{i}}\right)}^{2}$$

where $Y_{i}$ represents the observed target value, $\hat{Yi}$ is the predicted target value and *n* is the number of observations. *MSE* measures the average squared difference between the observed and predicted values, providing a sense of the prediction accuracy.

The Mean Absolute Error (*MAE*) is defined as:

(2) $$MAE=\frac{1}{n}\sum_{i=1}^{n}\left|Y_{i}-\hat{Y_{i}}\right|$$

where $Y_{i}$ represents the observed target value, $\hat{Yi}$ is the predicted target value and *n* is the number of observations. *MAE* measures the average absolute difference between the observed and predicted values, offering an easy-to-interpret measure of prediction accuracy.

The *R*-Squared (*R*^2^) is defined as:

(3) $$R^{2}=1-\frac{\sum_{i=1}^{n}{\left(Y_{i}-\hat{Y_{i}}\right)}^{2}}{\sum_{i=1}^{n}{\left(Y_{i}-\bar{Y}\right)}^{2}}$$

where $Y_{i}$ represents the observed target value, $\hat{Yi}$ is the predicted target value, and $\bar{Y}$ is the mean observed target value and *n* is the number of observations. *R*^2^ measures the proportion of variance in the observed data that is predictable from the independent variables, indicating the goodness of fit of the model.

In summary, these metrics (*MSE*, *MAE* and *R*^2^) provide comprehensive insights into the performance of the decision tree regression model by evaluating the accuracy and goodness of fit of the predictions.

### 4.2. Misclassification Issues in Decision Trees

Decision tree learning employs a divide-and-conquer strategy to identify optimal split points within a tree, iteratively classifying data records until achieving homogeneous subsets. While there exist various methods to choose the most suitable attribute at each node, two commonly used criteria in decision tree models are information gain and Gini impurity. These criteria aid in assessing the effectiveness of each test condition in classifying samples into respective classes.

Entropy, a fundamental concept in information theory, quantifies the impurity of sample values within a dataset. It ranges between 0 and 1, with zero indicating complete homogeneity and one indicating maximum entropy.

(4) $$Entropy(S)=-\sum_{c\in C}p\left(C\right)\log_{2}p\left(C\right)$$

where

- *S* represents the dataset that entropy is calculated for;
- *C* represents the classes in set, *S*;
- *p*(*C*) represents the proportion of data points that belong to class *C* to the number of total data points in set, *S*.

Information gain, on the other hand, measures the disparity in entropy before and after a split based on a specific attribute, aiming to identify the attribute that yields the most effective classification of training data.

(5) $$InformationGain(S,\alpha )=Entropy\left(S\right)-\sum_{v\in Values(\alpha )}\frac{|S_{v}|}{\left|S\right|}Entropy\left(S_{v}\right)$$

where

- α represents a specific attribute or class label;
- Entropy(*S*) is the entropy of dataset, *S*;
- $\frac{S_{v}}{S}$ represents the proportion of the values in $S_{v}$ to the number of values in dataset, *S*.
- Entropy($S_{v}$) is the entropy of dataset, $S_{v}$

Gini impurity quantifies the likelihood of misclassifying a randomly selected data point within the dataset if it were labeled according to the dataset’s class distribution. Like entropy, if the set *S* is pure (i.e., comprising only one class), its impurity equals zero, expressed by the following formula:

(6) $$GiniImpurity=1-\sum_{i=1}^{c}{\left(p_{i}\right)}^{2}$$

Getting the information on Gini impurity can possibly be correlated with the error in regressive prediction of absent sensor data samples, opening another option for error analysis.

We focus mainly on two of the machine learning prediction mechanisms based on decision trees here, one involving the random forest regression and another involving the gradient boosted trees.

#### 4.2.1. Random Forest Based Regression for Finding Missing IoMT Sensor Data

**Figure 3:**
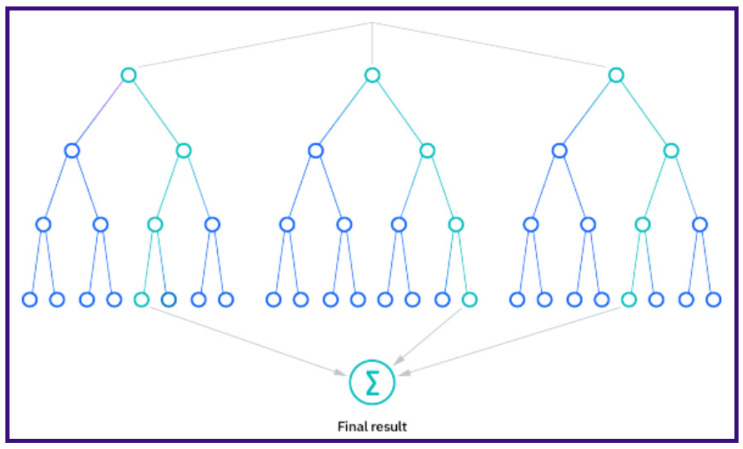
Graphical representation of random forest. Adapted from “What is random forest?”, by IBM, 2024, IBM. https://www.ibm.com/topics/random-forest (accessed on 22 May 2024).

The random forest algorithm relies on three primary hyperparameters, which must be configured prior to training: node size, the number of trees and the number of features sampled. Once set, the random forest classifier can be employed to address regression or classification tasks as depicted in Figure 3.

Comprising a collection of decision trees, the random forest algorithm constructs each tree within the ensemble using a data sample drawn from the training set with replacement, known as the bootstrap sample. Additionally, a subset of the training data, termed the out-of-bag (oob) sample, is reserved for evaluation purposes. Further diversification is introduced through feature bagging, minimizing correlations among decision trees. Prediction outcomes vary depending on the task type, with regression tasks averaging the predictions of individual trees and classification tasks employing a majority vote approach. The oob sample is subsequently utilized for cross-validation to finalize the prediction [64]. Figure 3 illustrates the process where the results of multiple decision trees are aggregated into a final outcome.

Random forest regression (RFR) is a machine learning technique that combines the principles of random forest with regression analysis. In RFR, multiple decision trees are constructed from randomly sampled subsets of the training data. Each decision tree independently predicts the target variable, and the final prediction is obtained by aggregating the predictions of all the trees. In the case of regression, the final prediction is typically the mean or median of the individual tree predictions. RFR is particularly useful for predicting continuous numerical outcomes, such as house prices, stock prices, or temperature. It offers several advantages, including robustness to overfitting, handling of high-dimensional data and the ability to capture non-linear relationships between variables.

#### 4.2.2. Gradient Boosting Based Regression for Predicting Missing IoMT Sensor Data

**Figure 4:**
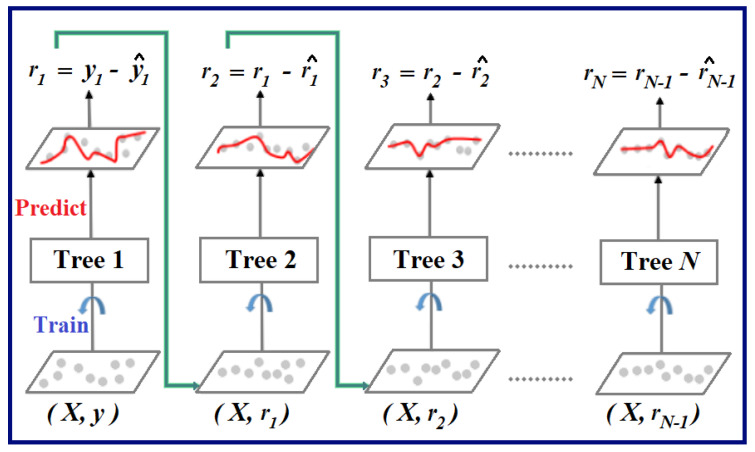
Graphical representation of Gradient boosted trees for regression. Adapted from “Gradient Boosting in ML”, by GeeksforGeeks, 2024, GeeksforGeeks. https://www.geeksforgeeks.org/ml-gradient-boosting/ (accessed on 22 May 2024).

Gradient boosting is a machine learning technique used for supervised learning tasks, such as classification and regression. It works by building a series of weak prediction models, typically decision trees, in a sequential manner. Each new model in the sequence corrects the errors of its predecessors, focusing on the areas where previous models performed poorly. This process is achieved by optimizing a loss function using gradient descent. The final prediction is a weighted sum of the predictions from all the models in the sequence as shown in Figure 4. Gradient boosting is known for its ability to produce highly accurate predictions and is widely used in various domains, including finance, healthcare and natural language processing.

In the context of regression, Gradient Boosted Trees form an ensemble comprising M trees [65]. The first tree, Tree1, is trained using the feature matrix X and labels *y*, producing predictions labeled *y*1(hat) used to compute the residual errors r1. Subsequently, Tree2 is trained with X and the residual errors *r*1 of Tree1 as labels, generating predicted results *r*1(hat) to determine residual *r*2. This iterative process continues until all M trees in the ensemble are trained (Figure 4 demonstrates this process). An essential parameter in this technique is shrinkage, where each tree’s prediction is scaled by a learning rate (eta) ranging from 0 to 1. A trade-off exists between eta and the number of estimators, necessitating a balance to achieve optimal model performance. With all trees trained, predictions are generated, where each tree predicts a label and the final prediction is computed accordingly. Each tree predicts a label and the final prediction is given by the formula,

(7) y(pred)=y1+(eta∗r1)+(eta∗r2)+⋯+(eta∗rN)

where

- y(pred) represents a predicted value;
- eta represents learning rate, ranging from 0 to 1;
- r1,r2,⋯,rN represents residual errors for Tree1, Tree2, …Tree*N* respectively;
- y1 represent the actual value;

Subjecting the samples of the arterial pressure and ECG signals after reduction to the prediction schemes involving decision trees and gradient boosting will result in some approximation that we have tried to analyze through calculations of mean square error.

## 5. Research Methodology

The authors have selected two physiological parameters for this research, the readings for which are contained in the data samples. The parameters are arterial pressure which is measured in millimeters of mercury column and ECG Lead II signal for which the measurement units are millivolts.

### 5.1. Handling the Reduction in the Amount of IoMT Data

For each reading, the authors removed different proportions of the original samples and reconstructed the signals at the smartphone end by applying a series of interpolation techniques to predict the missing values from the set. Two series consisting of the actual and predicted values were plotted and compared side by side and were used to consult with physicians and surgeons to determine the extent to which each reduced sample set could be counted on to reliably recreate the original physiological data. These sets corresponded to those missing exactly half, two-thirds and three-fourths of the original data. Error analysis of each of the interpolation techniques revealed that spline interpolation produced the better result across the various reduced sample sets. The authors have selected multiple sets of 3600 samples that encompass 10 s of sensor data for both ART and ECG signals in this research project.

The fidelity and accuracy of the data would rise if all of the samples of physiological sensor data were transmitted. The Nyquist criterion would essentially be met by such sampling as mandated, but it would also require conveying a large amount of data, but signal processing techniques can make this superfluous.

Implementing lossless compression methods on top of this encoding could result in even more efficient data compression. Data aggregation schemes have the potential to significantly reduce the transmission load of sensors.

Nevertheless, implementing such schemes may lead to a compromise between compression and fidelity. The level of error that can be tolerated in an approximation resulting from compressed encoding is a matter of debate, and the best judgment on the tradeoff should be made by physicians and specialists. The maximum error in encoding is decided by the step size and the maximum can be half the step size. For ART, the signal ranges from 50 mV to 90 mV, resulting in a signal span of 40 mV. When the signal is encoded into eight bits, the step size becomes 40 mV/256 = 156.25 µV, which in turn restricts the maximum permissible encoding error to 78.1 µV. The ECG lead-II signal error stands at 3.41 µV due to a signal-range from −0.75 mV to 1.0 mV, resulting in a step size of 6.83 µV. The findings for the two signals are outlined in Table 1.

The authors’ aim is to transmit physiological data through a 6G network utilizing two distinct methods. Prior to attempting to package IoMT data through a 6G network utilizing two distinct methods. Prior to attempting to package IoMT data into a text message, it is necessary to decrease the sample data volume by reducing the number of transmissions. The application of signal processing techniques can help predict missing sample values within acceptable error limits, thereby reducing the number of samples in the data. Consultation with the end users and getting their opinion, who in this scenario are the cardiac physicians and surgeons, is necessary to determine the acceptability of the level of approximation. Under no circumstances should the approximation impact or change their diagnosis. Throughout the process, we adhered to this procedure and sought guidance from the cardiologists at every step when conducting these estimations. Mishra et al.’s dual prediction techniques [55] can also be applied at the receiving end to reconstruct missing samples, in addition to the method discussed in our article. We minimized the data volume by excluding samples from the initial dataset that can be anticipated at the destination.

Our proposed system primarily emphasizes the real-life ART and ECG signals associated with both healthy individuals and actual patients, which were acquired from Physionet.org.

#### Reconstruction of Signals Post Reduction of Samples

The signal range specifications for the two signals are displayed in Table 2. Upon examination of Table 2, it is apparent that the ART signal has the wider range of approximately 50.0 mV, while the ECG Lead—II signal has the narrower range at about 1.75 mV.

The sample data from the signal datasets were transformed into four distinct subsets for each original set, with a gradual decrease in the number of samples in the signal.

The initial subset consisted solely of the alternate samples, while the subsequent subset encompassed every third sample. The third set consisted of every fourth sample, and ultimately, the fourth set consisted of every fifth sample. As a result, the first, second, third and fourth sets had sample sizes that were half, a third, a fourth and a fifth of the original set, respectively. The four sets were subsequently sent and received on the opposite side. At the receiving terminal, the initial set of samples was reconstructed in its entirety through numerical interpolation using these four sets. The missing samples that were reconstructed were subsequently analyzed against the initial complete sample set to identify any discrepancies between the two sets. The outcomes of the reconstruction following sample reduction are displayed in the following figures for the ECG-Lead II signal and the ART signal.

**Figure 5:**
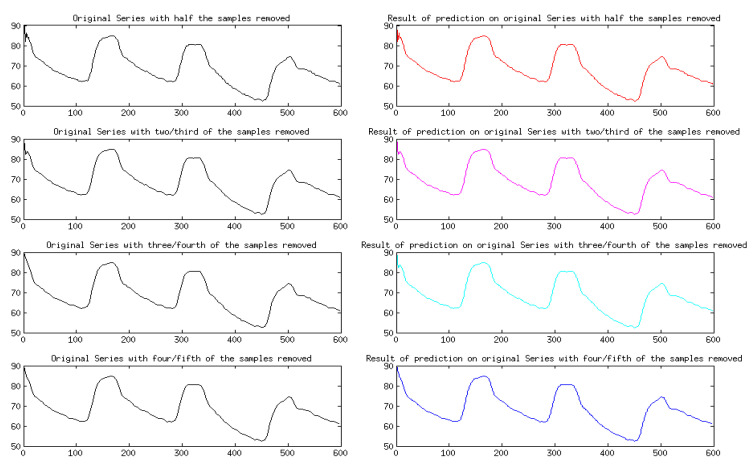
Signal recreation through prediction for ART signal for different sample frequencies.

A comparison was made between the recreated signals and the original sets, and an error analysis is provided in Table 3. Based on Table 3, it is evident that the error is lower for the ART signal samples in comparison to the ECG signal. The reason for this is that the ECG signal has a wider range and different wave nature, which results in more intricate and nuanced variations. These variations are encoded with a smaller step size compared to the ART signal. Figure 5 shows the results only for four cycles of the data for the ART signal.

The difference in aggregation method between our two regression methods influenced the final shape of our research. Not only did we wish to calculate values for different ratios of a dataset, but we equally sought to understand how the number of trees, n, would impact the relative accuracy of each regression model. Consequently, our research consisted of performing a series of statistical regression tests on two sets of data values pulled from Mishra & Agrawal [10]. These were selected for producing distinct sinusoidal patterns that closely mimic those of other subsets recorded with the same unit of measurement: The first was the electrocardiogram data (ECG) pulled from the “ECG Lead II column” that recorded millivolts (mV) over time, and the second was the ART column that recorded blood pressure in milligrams of mercury (mmHg) over time.

The statistical analysis would take two forms of variation. In the first form, we removed the set ratios of data points from the original dataset of 3600 records to assess each regressor’s efficiency in replicating the observed values; each regression result will then be graphed side-by-side with the original column set for visual clarity. These ratios took the form of half data removed, two-thirds of data removed, three-fourths data removed with the full dataset serving as our control group. In addition, $R^{2}$ scores and mean squared errors were calculated to estimate the closeness of fit between the actual and predicted results. In the second form of variation, the aforementioned procedure was repeated three times using the following number of decision trees: 10, 100 and 1000. In doing so, we hoped to ascertain the general effectiveness that increasing the number of decision trees has on each regression algorithm.

The coefficient of determination determines the overall fit between the actual values and the predicted values by returning a number between 0 and 1, inclusively as proposed by Raschka et al. [66]; the closer the number is to 1, the greater the fit. Contrarily, a value of −1 would indicate that the model has become defective. The coefficient of determination suffers from one major deficit in that it can leave out key information about outliers; however, this should not concern our analysis as the only outliers present found in the ART data are byproducts of the measuring tool and thus do not veritable cardiac activity. Mean squared error, meanwhile, represents the averaged square difference between the actual and predicted values of the regression model [66]. The smaller the error, the closer the predicted values are to the actual values.

### 5.2. Statistical Tools Used for This Research

All programs were written in Python using Jupyter Notebooks with the scikit-learn’s free machine learning library geared towards data analysis that was used to conduct the regression analyses and Matplotlib to visualize the results. In addition, the authors used Python’s in-built algorithm from its native random package to generate random datasets in each round of analysis.

**Figure 6:**
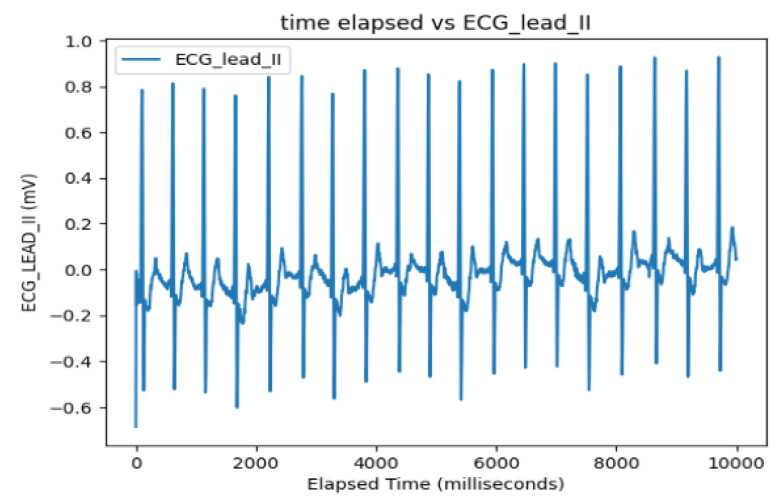
ECG Lead-II: Full dataset.

**Figure 7:**
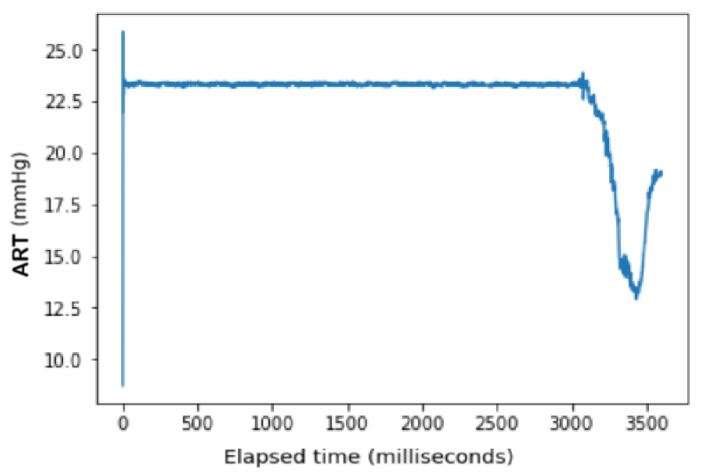
ART Data: Full dataset.

It should be noted, however, that one major adjustment was required prior to conducting the linear regression models. Whereas the ECG Lead II data display a consistent pattern throughout the length of the data span, the recording for ART shows a period of logarithmic growth and decay at the beginning prior to stabilizing to a consistent pattern before trailing off at around t = 3000 (Figure 6 and Figure 7). This irregular behavior is attributed to the nature of the measuring tool used to measure arterial pressure which undergoes an initial setup period that is not reflective of actual cardiac activity. As a result, trimming this outlier data resulted in sample sizes of 3010 compared to the full 3600 data points used to measure the electrocardiogram activity.

## 6. Results

The ECG data display a recurrent pattern that repeats itself over a time period of approximately 200 milliseconds characterized by a parabolic rise to a little less than 0.2 millivolts in the first 50 milliseconds, followed by a brief pause wherein the voltage hovers closely to 0 milliseconds before exploding up to 0.2 volts before dropping to approximately −0.6 millivolts, after which it repeats the same pattern (see Figure 6). In contrast, the rise and falls seen in the ART data are far more consistent in their shape, with most of the data limited to a range between 23.20 volts and 23.40 volts, while the changes occur over a far shorter time span (see Figure 7).

**Figure 8:**
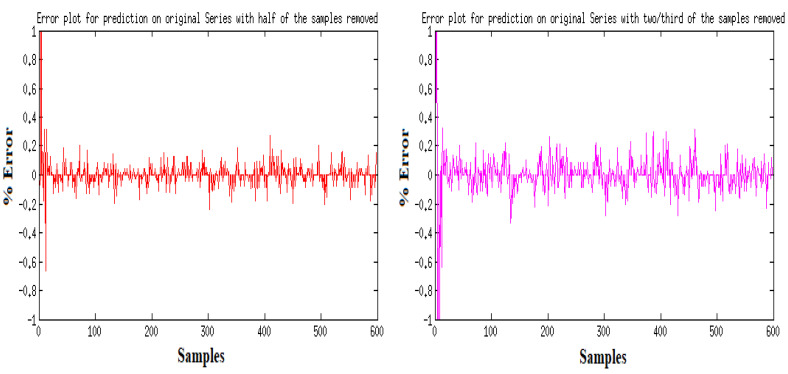
Error in prediction for ART Data with reduced samples: full dataset.

**Figure 9:**
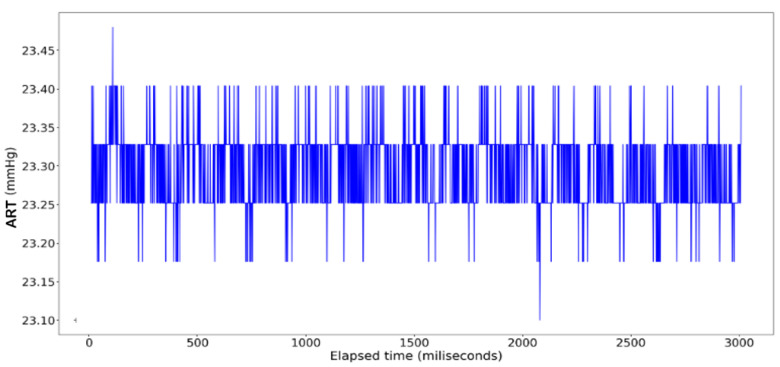
ART Data: dataset with outliers removed.

**Figure 10:**
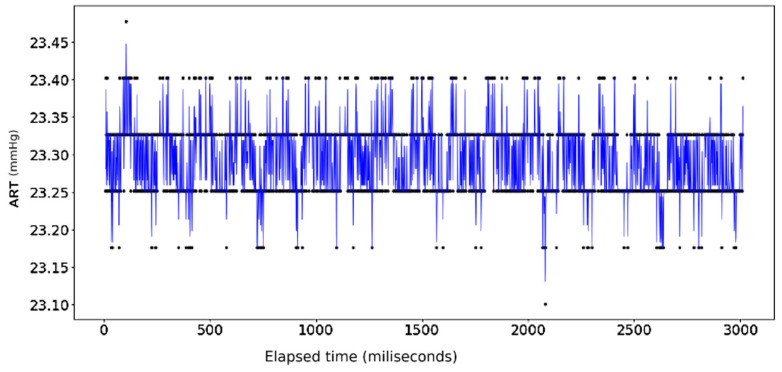
ART Data: full dataset (*n* = 10).

The results of the first round of regressions with *n* = 10 decision trees show high fidelity on the part of the random forest models. The error in prediction for the ART signal samples is displayed in Figure 8. Figure 9 shows the full dataset of the ART signal across the dataset. The waveform in Figure 10 displays the predicted ECG values in colored lines vis-à-vis the actual values, plotted as black dots, at different ratios of the original data. At each ratio the predicted values retain the same shape as that of the actual values and this closeness of fit is confirmed by the $R^{2}$ scores and errors, with the former calculated at over 0.99 while the latter consistently stays below 0.15. The model retains its fidelity in the subsequent iterations as the number of trees incorporated into the model is increased to 100 and 1000, exhibiting $R^{2}$ scores and errors that are nigh indistinguishable from those made from the *n* = 10 model (see Table 4).

**Figure 11:**
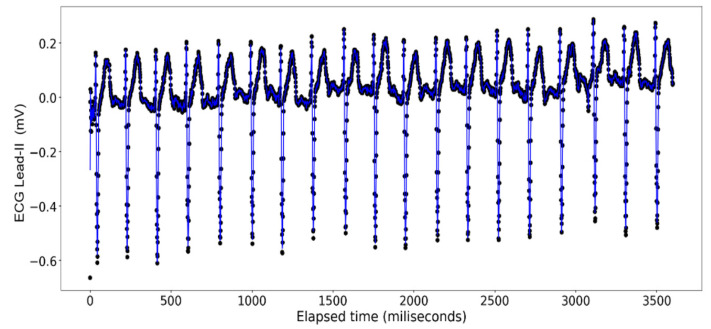
Random Forest Regression on ECG Lead-II Data: full dataset (*n* = 10).

**Figure 12:**
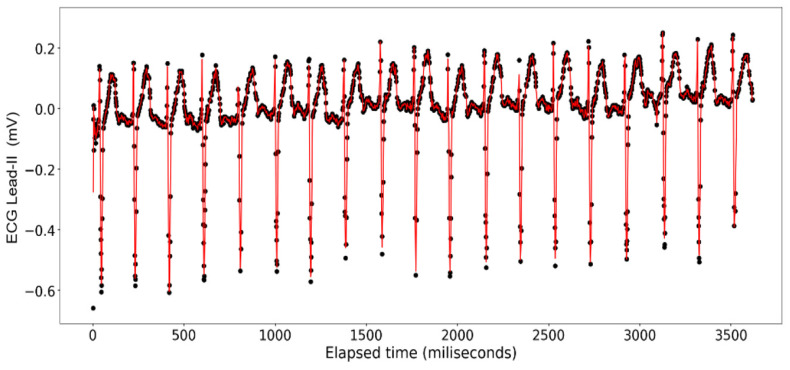
Random Forest Regression on ECG Lead-II Data: half data removed (*n* = 10).

**Figure 13:**
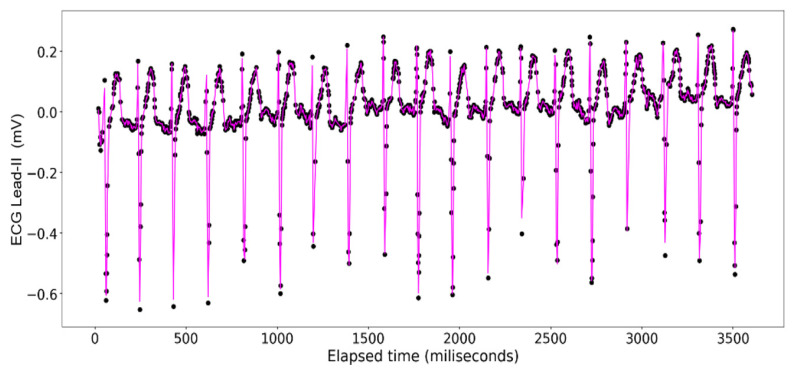
Random Forest Regression on ECG Lead-II Data: two-third data removed (*n* = 10).

**Figure 14:**
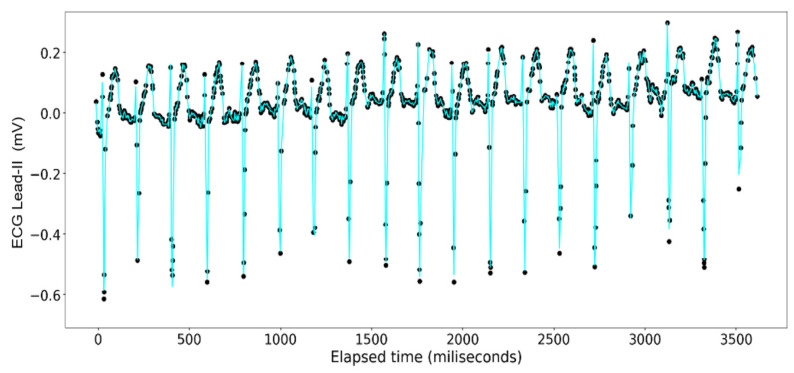
Random Forest Regression on ECG Lead-II Data: three-fourth data removed (*n* = 10).

The Figure 11, Figure 12, Figure 13 and Figure 14 show the results of application of Random Forest regression for signal reconstruction for the ECG lead-II signals with *n* being 10.

**Figure 15:**
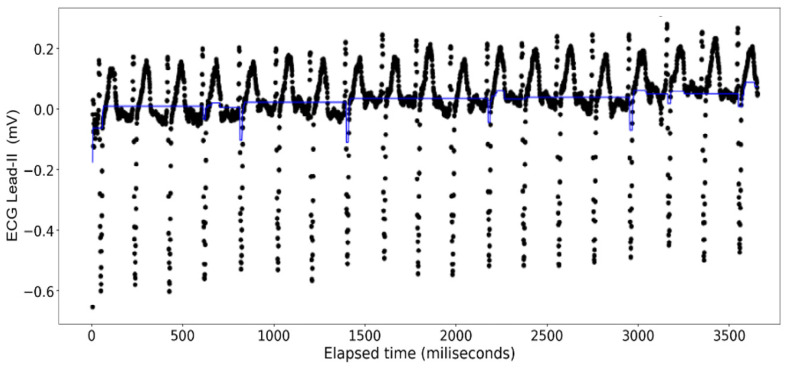
Gradient Boosting Regression on ECG Lead-II Data: full set (*n* = 10).

**Figure 16:**
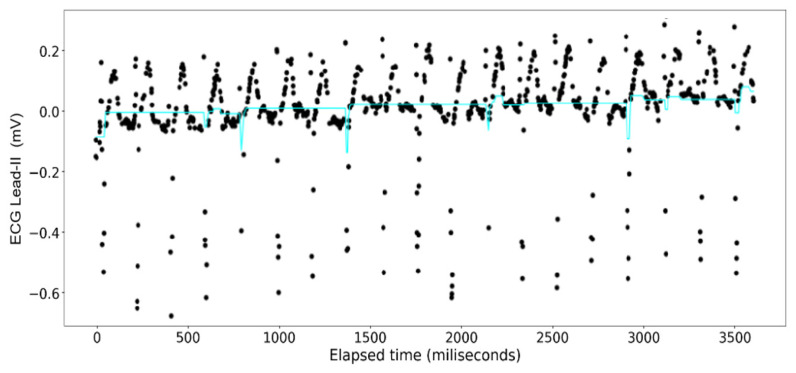
Gradient Boosting Regression on ECG Lead-II Data: three-fourth data removed (*n* = 10).

**Figure 17:**
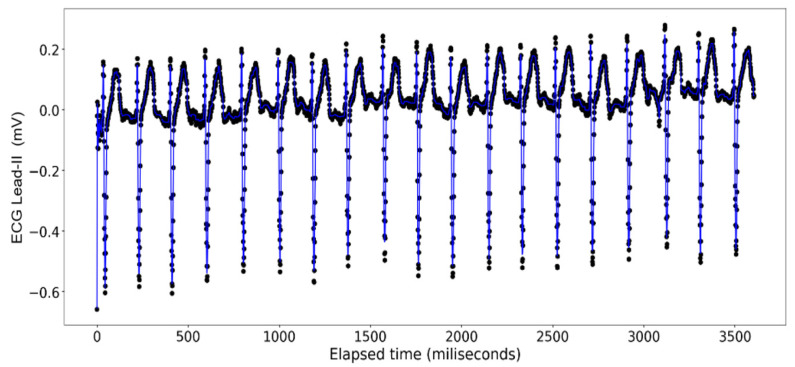
Gradient Boosting Regression on ECG Lead-II Data: full set (*n* = 1000).

**Figure 18:**
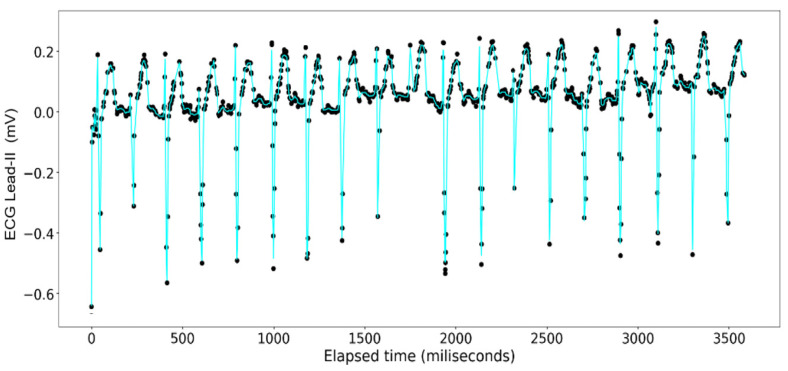
Gradient Boosting Regression on ECG Lead-II Data: three-fourth data removed (*n* = 1000).

The Figure 15, Figure 16, Figure 17 and Figure 18 show the results of application of Gradient Boosting regression for signal reconstruction for the ECG lead-II and with *n* being 10 and 1000 with some samples removed. There are similar results for *n* = 100 for the ECG signal with different rates of samples removed that are not shown in these figures. Results for the ART signal were also collected for all these combinations but have not been shown in this article but included in the summary and findings further ahead.

When the gradient boosting algorithm was run for *n* = 1000, following results were received for the ECG Lead—II signal.

**Figure 19:**
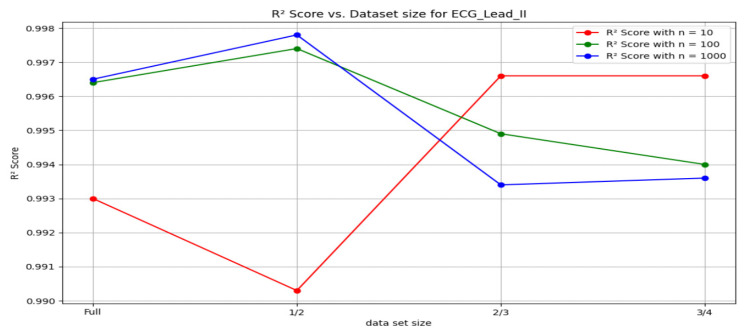
$R^{2}$ scores v/s Dataset size for ECG-Lead II signal.

Figure 19 shows the behavior of $R^{2}$ scores against the size of the dataset used for the ECG—lead II samples plotted for the different *n* values in the method involving random forest regression.

**Figure 20:**
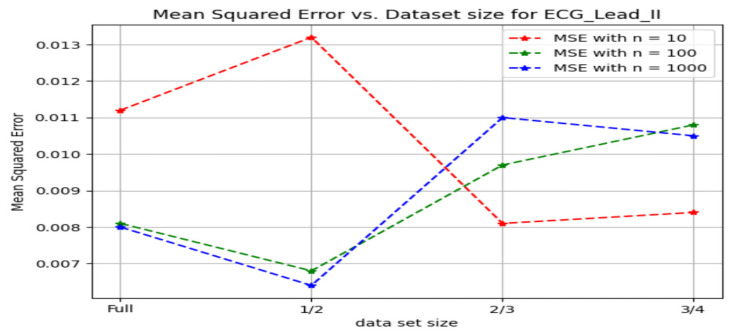
Mean square error v/s dataset size for ECG Lead—II.

Figure 20 shows the changes in the mean squared error marked against the size of the dataset used for the ECG—lead II samples plotted for the different *n* values in the method involving random forest regression.

An initial analysis of the $R^{2}$ scores and Mean Squared Errors ((MSE) for various dataset sizes and numbers of trees (n) in the Random Forest algorithm revealed an intricate interplay. When *n* = 10, the full dataset achieved an $R^{2}$ score of 0.993, which marginally decreased to 0.990 for half the dataset size. However, this decrease in $R^{2}$ score coincided with a significant increase in (MSE, indicating a poorer fit for the reduced dataset. Interestingly, the $R^{2}$ scores for two-thirds and three-quarters of the dataset size exhibited near equivalence, even surpassing the full and half dataset scores. However, the (MSE for the three-quarters dataset was higher than for the two-thirds dataset, suggesting a potential overfitting issue. In contrast, *n* = 1000 yielded the highest $R^{2}$ score (0.9978) for the half dataset size, accompanied by a lower (MSE compared to all other sizes. While *n* = 100 also demonstrated a high R^2^ score for the half dataset, it fell short of the peak achieved by *n* = 1000 in terms of $R^{2}$ and exhibited a slightly higher (MSE. In conclusion, these observations suggest a potential sweet spot for *n* = 10 when using two-thirds or three-quarters of the dataset, balancing $R^{2}$ score and avoiding overfitting as indicated by (MSE. Conversely, *n* = 1000 appears optimal for the half dataset size, achieving the highest $R^{2}$ score with a demonstrably lower (MSE. Further investigation is warranted to elucidate the underlying mechanisms governing these interactions and identify potential strategies for optimizing hyperparameter selection across varying dataset sizes.

The results of the random forest model’s performance with the ART data show a radical digression from those of the ECG shown in the Table 4. In this case, the closeness of fit can only be seen in the graphs wherein the model uses the entire dataset of arterial pressure to calculate the $R^{2}$ scores and errors that are consistent with those calculated ECG Lead II. More interesting, once the random forest model is used on the subsequent decreasing ratios of the ART data, model returns negative values as shown in the table, implying that the model can no longer yield results that would be more accurate if the $R^{2}$ score and mean squared error had been calculated using simple linear regression.

Analysis done with the gradient boosting algorithm on the ECG data show a poor fit across varying sizes of the original data when the number of decision trees used is set at 10. Contrary to the random forest model, however, the overall closeness of fit clearly increases from one iteration to another (see Figure 16 and Figure 17) where at 1000 decision trees the graphs look indistinguishable from those made with the random forest regression. This closeness of fit is reinforced by the $R^{2}$ scores, and the errors calculated on Table 5.

**Figure 21:**
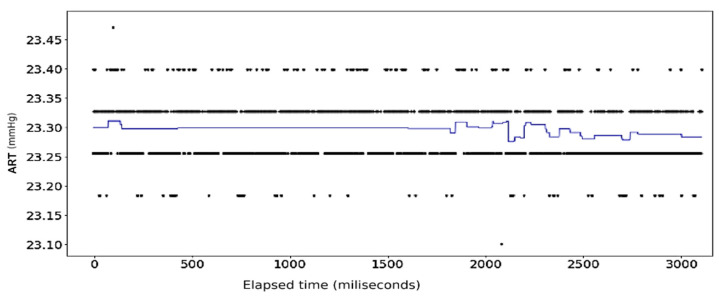
Gradient Boosting Regression on ART Data: full set (*n* = 10).

**Figure 22:**
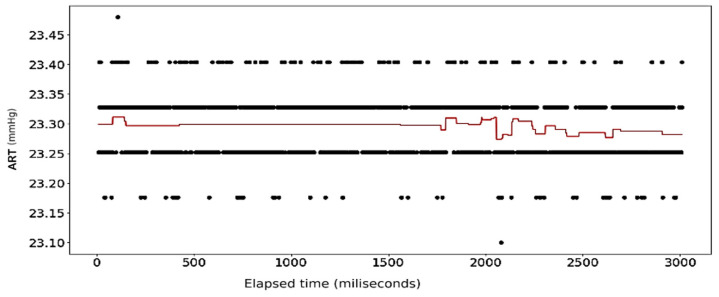
Gradient Boosting Regression on ART Data: half data removed (*n* = 10).

**Figure 23:**
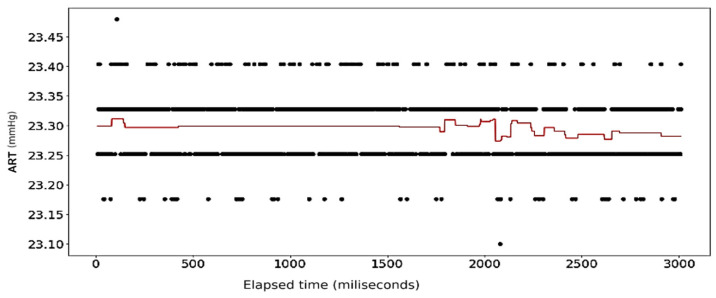
Gradient Boosting Regression on ART Data: half data removed (*n* = 100).

**Figure 24:**
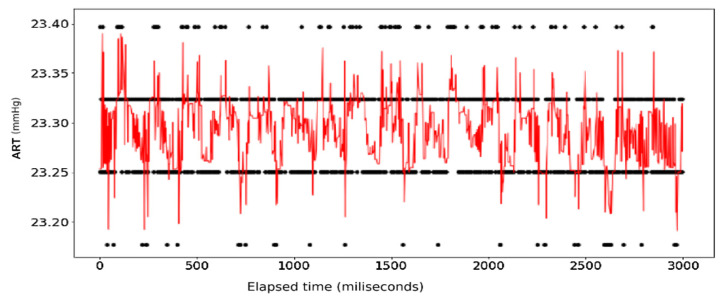
Gradient Boosting Regression on ART Data: half data removed (*n* = 1000).

Figure 21, Figure 22, Figure 23 and Figure 24 show the results of application of Gradient Boosting algorithm on the ART data. Figure 22 and Figure 23 look strikingly similar with the limited resolution here but are not the same.

**Figure 25:**
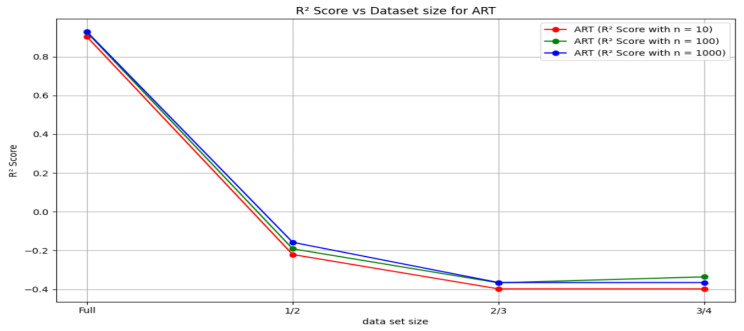
$R^{2}$ scores v/s Dataset size for ART signal.

Figure 25 shows the behavior of $R^{2}$ scores against the size of the dataset used for the arterial pressure samples plotted for the different *n* values in the method involving random forest regression.

**Figure 26:**
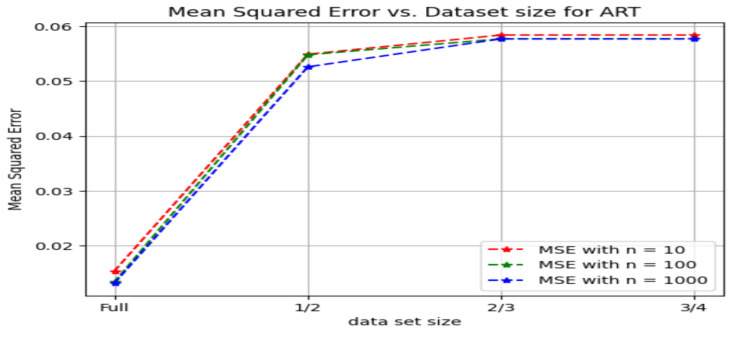
Mean square error v/s dataset size for the ART signal.

Figure 26 shows the changes in the mean squared error marked against the size of the dataset used for the arterial pressure samples plotted for the different *n* values in the method involving random forest regression.

Interestingly, when the gradient boosting regressions were performed on the full sample of the arterial pressure readings, we observed a similar gradual improvement on the fit of the graph; however, this improvement fell short of matching the closeness of fit that was observed with the random forest regression as indicated in Table 6. When it came to reducing the size of the physiological samples, only the models that utilized 100 and 1000 decision trees produced a drop in the fit. On the other hand, the model with 10 decision trees saw a slight boost, although unremarkable, when reducing half of the data sample. Finally, once the number of decision trees was set to 1000, the gradient boosting algorithm could no longer produce reliable $R^{2}$ scores, returning negative values instead.

We discuss the possible reasons for these changes in the following section.

## 7. Discussion

Results confirm the potential for improving the effectiveness of using IoMT networks in transmitting real-time data to medical professionals over previously existing algorithms derived from pure linear regression. More specifically, they demonstrate the power of random tree-derived models and illustrate how taking one or more different approaches towards predicting data values can benefit depending on the situation, particularly the shape of the graph. Our first approach using the random forest model takes the sum of all decision trees to predict a value. Results from the analysis on the electrocardiogram readings suggest that forest regression has tremendous predictive powers on sets with distinct repeating sinusoidal patterns that can occur over a duration of at least 200 milliseconds regardless of the number of decision trees that are implemented when constructing.

### 7.1. Performance Summary for the Random Forest Regression Methods

The predictive potential of random forest regression for use in signal transmission is perhaps best illustrated by comparing the error rates with the linear regressions used in Mishra and Agrawal in [10], as seen in Table 7. Regardless of the number of decision trees implemented, the % error rates for the predicted values generated by the random forest regeneration undercut those acquired through linear regression by a factor of at least 17.5%, which is a considerable improvement.

### 7.2. Performance Summary for the Gradient Boosting Decision Method

Our second approach using the gradient boosting algorithm predicts a missing value while gradually building and improving its decision algorithm from the result of the first decision tree and continues to do so until the last decision tree has executed its algorithm. Contrary to the random forest regression, however, this approach’s efficiency is dependent on the number of trees and therefore could be considered less desirable than its random forest counterpart. In addition, its performance varies more when we begin subtracting the number of data points available to the model, a fact that became apparent when comparing the performance of both models with the arterial pressure readings. Whereas the closeness of fit dropped quite noticeably when the dataset was reduced to half of its original numbers, the $R^{2}$ scores of the random forest trials turned negative, a fact that indicates that the model no longer performs better than using the simpler linear regression for predicting missing values.

## 8. Conclusions

The work presented here is an extension of research work generated out of previous research by the lead author. The first one of the two ideas is the use of the two techniques in detecting, predicting and filling up erroneous or missing data that have been deliberately introduced by the authors in the datasets for energy saving and improving the network lifetime as the sensors are mostly powered by batteries. Such gaps in data could also happen as a result of sensor failure, or a cyber-attack. The authors have presented the summarized results from the applications of the two broad techniques and the parametric changes while applying the techniques. The authors have also compared the results with other techniques previously used by the lead author and tried to explain the results in the later sections. The present analysis focuses on the approximation error resulting from the applications of the two prediction techniques as a basic evaluation of the efficacy of the new applied techniques and a comparison of errors from previously tested techniques.

Both the gradient boosting regression and random forest regression represent two powerful tools for expanding the potential of preventative medicine, providing high quality physiological results to medical professionals. And while they represent powerful statistical tools over traditional linear regression, our results have been shown that the random forest tree possesses several advantages over the gradient boosting algorithm in what concerns predicting missing values in physiological datasets. Most important is that the random forest regression model produces strong approximations to the original missing data while needing fewer decision trees in its construction and losing no significant degree of predictive potency even when stripping it up to three-quarters of a data of 3600 points.

Nonetheless, it was equally important to notice that the applicability of decision tree-dependent algorithms is limited, not to the quantity of data available or the number of decision trees it is built on, but to the very trend of the data available. In datasets where there is an asymmetrical distribution of the data around a central horizontal axis, as seen with the electrocardiogram measurements, the random forest decision tree was far more accurate in replicating missing data as any linear function would only produce a sub-optimal fit.

However, the case was the opposite when applying the models to the arterial pressure where the data were distributed in such a way that the overwhelming majority of points fell among two sets of y-values. This observation is illustrated in Figure 3, which graphs the regression results of the gradient boosting algorithm done at each model iteration on the halved datasets. The entire dataset is contained within two sets of y-values: an inner set with values at around 23.33 and 23.25 containing over 50% of all data values, and an outer set with values of 23.40 and approximately 23.18 containing over 90% of the data. Whereas the 1000 tree model produces a shape that is more reminiscent of the full dataset, the resultant $R^{2}$ score is actually less than the graphs produced with only 10 and 100 trees. These graphs display a prolonged section from t = 100 to t = 1800 that is quasi-linear which suggests that, overall, the shape of the graph, in addition to other parameters such as the number of decision trees utilized and the size of the pool of actual values, needs to be taken into consideration when selecting a regression method for predicting datasets. Consequently, linear models should not only be used when analyzing linear unidirectional data but also with data that are heavily symmetrical around a fixed y-value, specifically one wherein a sinusoidal shape cannot be made out.

## 9. Further Study

The results of this experiment underline the potential for using decision tree-derived regression, more specifically that of random forest regression, for producing more accurate physiological signals remotely gathered and delivered to health care professionals via the use of IoMT networks. What remains now is to design experiments that can harness their power. Whereas this proved to be a limitation in the cited research articles, the continuous, rapid growth in wireless technologies make it now possible to transmit larger and more accurate segments of data by taking advantage of the higher computing power of wireless devices and the increased bandwidth in wireless networks.

Whereas the cell phones used until a few years back as coordinating sink stations (CSS) possessed only a single core processor, the latest smartphones, as of the date of publication of this article, can hold up to 5-core and 4-core processors. Moreover, the recent and continuing unfurling of 5G networks in the last five years provides data rates with peak rates greater than 1 gigabits per second and latencies of 10-1 milliseconds, which far outpace the 42 megabit per second peak rate and 100 millisecond latency of the previous 4G generation technology as given by Mishra [67]. In addition to wireless networks, studies could make equal use of cellular data networks, Bluetooth, Zigbee and 6LowPAN networks for relaying physiological data.

Finally, the CSS unit should not only have resources for hosting more complex regression algorithms, but be capable of running algorithms that can determine, given the signal input and the function parameters set by the experimenter, whether the situation benefits from using the linear regression over that of the more complex random forest regression. Depending on the system parameters of the IoMT architecture, it could be equally worthwhile to investigate using other decision-trees algorithms which we could not cover in our experiments, such as the simpler decision-tree algorithm.

Additionally, delta encoding can be implemented on top of the proposed architecture to enhance the compression of information within each message packet carrying encoded IoMT data, which we propose to take up as future work.

## Figures and Tables

**Table 1:** Signal specifications for the two vital sign IoMT parameters.

Characteristics	ART	ECG-II
Signal Span	40.0 mmHg	1.75 mV
Encoding Error	0.078 mmHg	3.41 µV
Step Size	0.156 mmHg	6.83 µV

**Table 2:** Signal ranges for the two vital sign IoMT parameters.

Characteristics	ART	ECG-II
Signal Minimum	50.0 mmHg	−0.5 mV
Signal Maximum	90.0 mmHg	1.0 µV
Signal Span	40.0 mmHg	1.5 µV

**Table 3:** Post sample cut maximum error in prediction for ART & ECG Lead II signals.

Signal	Parameter	1/2	1/3	1/4	1/5
ART	Error	0.27 mV	0.32 mV	0.39 mV	0.63 mV
	%	0.72	0.85	1.05	1.69
ECG Lead-II	Error	0.05	0.08	0.13	0.20
	%	3.77	6.15	9.67	15.00

**Table 4:** $R^{2}$ scores and mean squared errors for the Random Forest Regression on ECG Lead II.

*n*	Result	Full	1/2	2/3	3/4
*n* = 10	$R^{2}$	0.993	0.9903	0.9966	0.9966
	Error	0.0112	0.0132	0.0081	0.0084
*n* = 100	$R^{2}$	0.9964	0.9974	0.9949	0.994
	Error	0.0081	0.0068	0.0097	0.0108
*n* = 1000	$R^{2}$	0.9965	0.9978	0.9934	0.9936
	Error	0.008	0.0064	0.011	0.0105

**Table 5:** Random Forest Regression on the ART signal: $R^{2}$ scores and mean squared errors for various sample rates.

*n*	Result	Full	1/2	2/3	3/4
*n* = 10	$R^{2}$	0.9031	−0.2207	−0.3979	−0.3979
	Error	0.0154	0.0549	0.0584	0.0584
*n* = 100	$R^{2}$	0.9259	−0.1913	−0.3667	−0.336
	Error	0.0134	0.0548	0.0577	0.0577
*n* = 1000	$R^{2}$	0.9286	−0.1578	−0.3653	−0.3653
	Error	0.0132	0.0526	0.0577	0.0577

**Table 6:** Gradient Boosting Regression on the ART signal: $R^{2}$ scores and mean squared errors for various sample rates.

*n*	Result	Full	1/2	2/3	3/4
*n* = 10	$R^{2}$	0.0508	0.0796	0.0736	0.0736
	Error	0.0485	0.0477	0.0475	0.0475
*n* = 100	$R^{2}$	0.3207	0.1999	0.1844	0.1844
	Error	0.0407	0.0449	0.0446	0.0446
*n* = 1000	$R^{2}$	0.7174	0.0993	−0.0192	−0.0192
	Error	0.0263	0.0464	0.0499	0.0499

**Table 7:** Arterial pressure: Error % with different rates of sample reduction.

Technique	Parameter	1/2	2/3	3/4
Numerical Interpolation	-	0.72	0.85	1.05
Random Forest Regression	*n* = 10	0.05	0.06	0.06
	*n* = 100	0.05	0.06	0.06
	*n* = 1000	0.05	0.06	0.06

## Data Availability

The data presented in this study are available on request from the corresponding author.

## Abbreviations

The following abbreviations are used in this manuscript:

5G	Fifth Generation Cell phone and data network
6G	Sixth Generation Cell phone and data network
A3C	Asynchronous Advantage Actor-critic
AI	Artificial Intelligence
ART	Systemic Arterial pressure
CSS	Coordinating Sink Station
ECG/EKG	Electrocardiogram lead II
HMU	Health Monitoring Units
IoMT	Internet of Medical Things
LPU	Local Processing Unit
MEMS	Micro Electro-mechanical Systems
MIMO	Multi Input Multi Output
mmHg	Millimeters of Mercury Column
NEE	Net Ecosystem carbon Exchange
OGWO	Oppositional Grey Wolf Optimization
OOB	Out of Bag
RFR	Random Forest Regression
R(MSE	Root Mean Square Error
WSN	Wireless Sensor Networks
